# Supplementary material for: An active site loop toggles between conformations to control antibiotic hydrolysis and inhibition potency for CTX-M β-lactamase drug-resistance enzymes
Source: Nat Commun. 2022 Nov 7;13:6726. doi: 10.1038/s41467-022-34564-3 (PMC9640584; doi:10.1038/s41467-022-34564-3)
Supplement: Supplementary file 1 — Supplementary Information [file 41467_2022_34564_MOESM1_ESM.pdf]

SUPPLEMENTARY INFORMATION

An Active Site Loop Toggles Between Conformations to Control Antibiotic Hydrolysis and Inhibition Potency for CTX-M β-lactamase Drug-Resistance Enzymes

Shuo Lu, Liya Hu, Hanfeng Lin, Allison Judge, Paola Rivera, Murugesan Palaniappan, Banumathi Sankaran, Jin Wang, B.V. Venkataram Prasad and Timothy Palzkill

Supplementary Figures

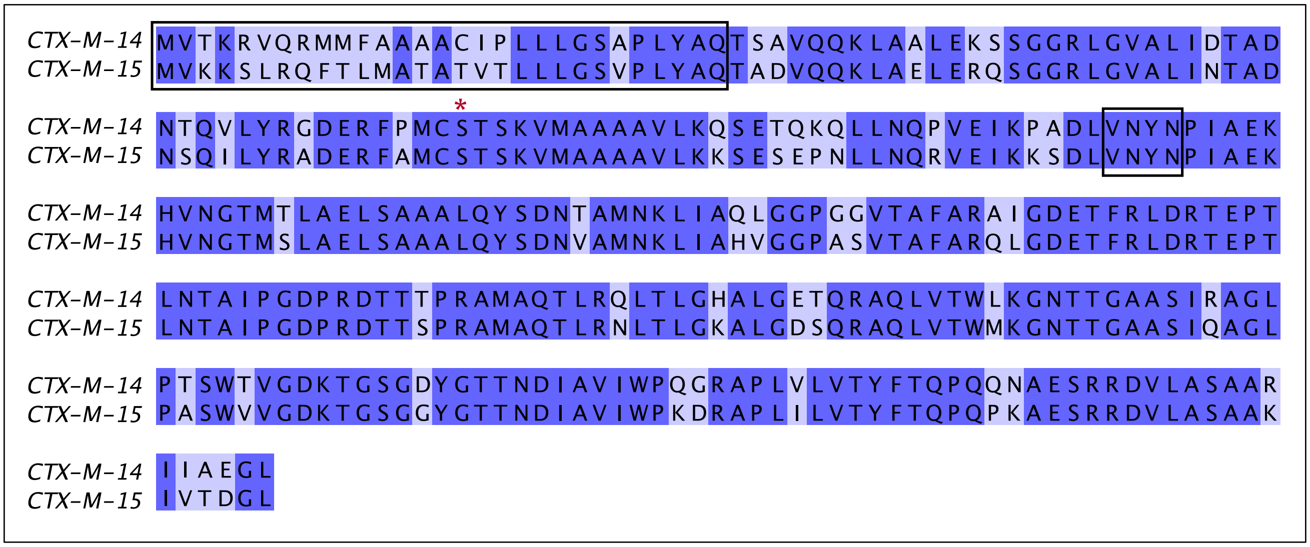

Supplementary Figure 1.
Amino acid sequence alignment between CTX-M-14 and CTX-M-15 β-lactamases. The signal sequence and the V103-N106 loop regions are boxed with black lines. Identical amino acids between the sequences are colored dark blue while amino acid positions that differ between the enzymes are in light blue. The catalytic Ser70 residue is indicated with a red asterisk.

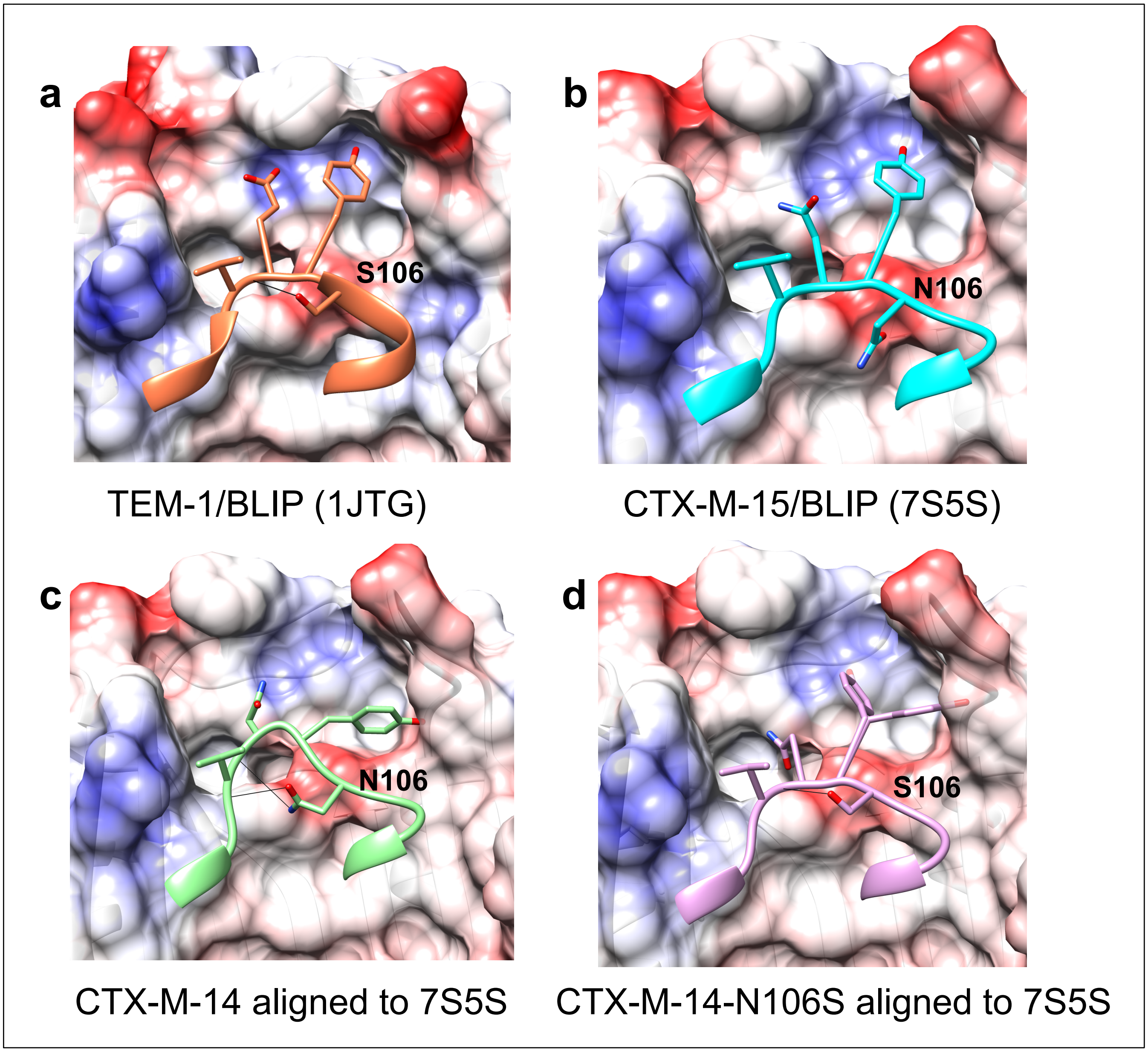

Supplementary Figure 2.
β-lactamase 103-106 loop in complex with BLIP. **a** Structure of TEM-1 β-lactamase in complex with BLIP (pdb id: 1JTG). β-lactamase residues 101-109 are shown in ribbon (orange). Ser106 is labeled and the hydrogen bond from Ser106 to the Val103 main chain is shown as a black line. **b** Structure of CTX-M-15/BLIP (pdb id: 7S5S). β-lactamase residues 101-109 are shown in ribbon (cyan). Asn106 is labeled. **c** Structural alignment of CTX-M-14 to the CTX-M-15/BLIP structure. CTX-M-14 residues 101-109 are shown in ribbon (green). Asn106 is labeled and the hydrogen bonds from Asn106 to the Val103 main chain are shown as black lines. **d** Structural alignment of CTX-M-14 N106S mutant to the CTX-M-15/BLIP structure. CTX-M-14 N106S residues 101-109 are shown in ribbon (pink). Ser106 is labeled and the hydrogen bond from Ser106 to the Val103 main chain is shown as a black line.

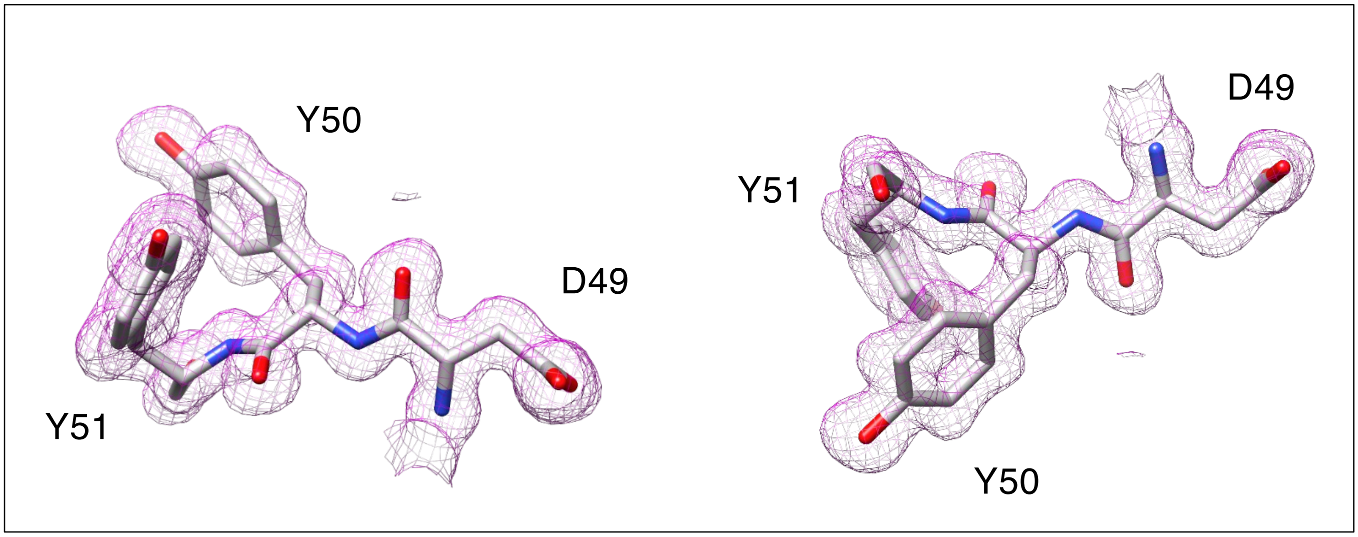

Supplementary Figure 3.
Electron density (Polder) map of BLIP residues Asp49-Tyr51 from the BLIP/CTX-M-15 β-lactamase structure at 3.0σ, showing the BLIP loop positioning within the election densityfrom two different angles.

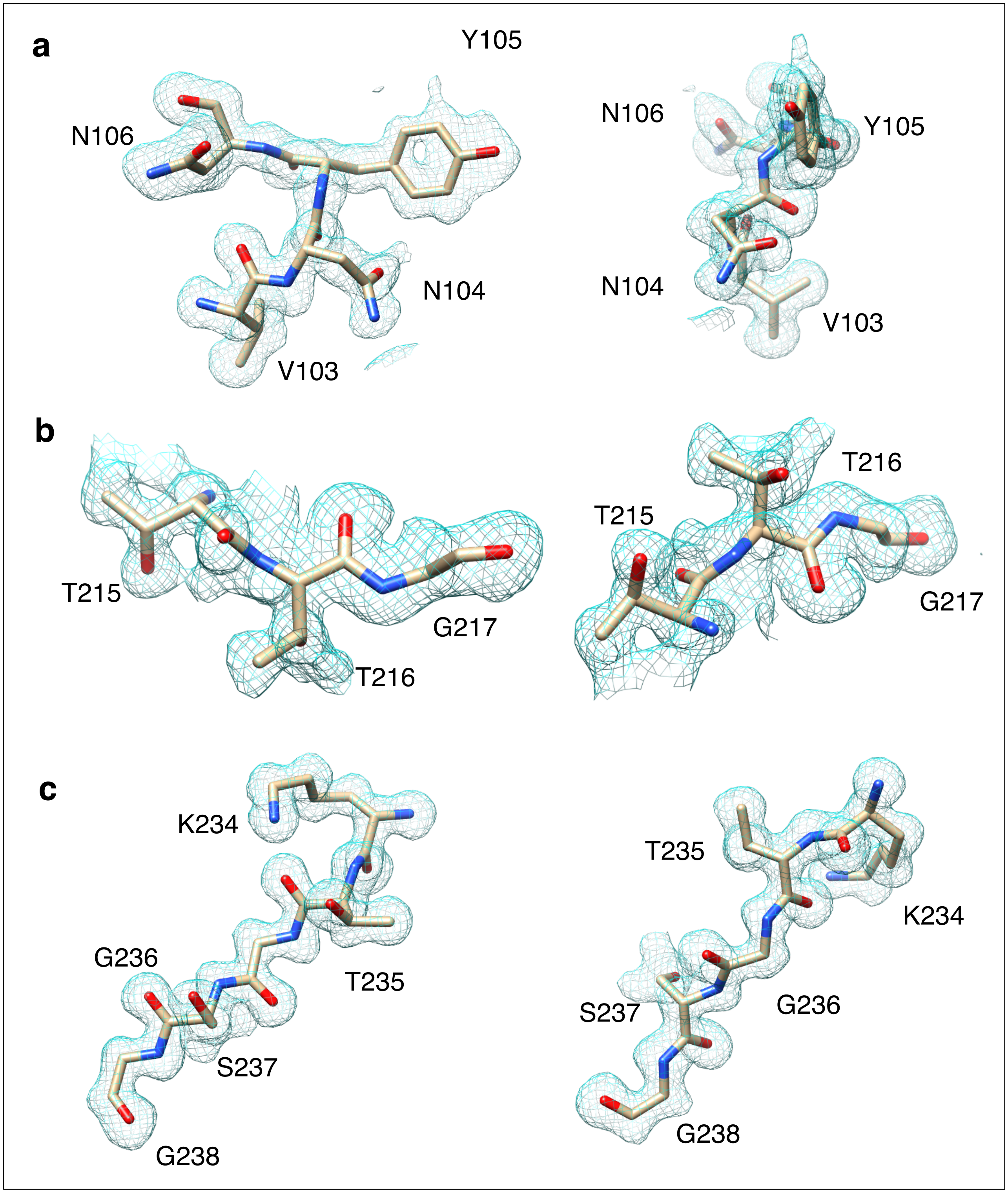

Supplementary Figure 4.

Electron density (Polder) map from the BLIP/CTX-M-15 b-lactamase structure at 3.0σ, constructed with CTX-M-15 residues, **a** Val103-Asn106, **b** Thr215-Gly217, and **c** Lys234-Gly238, which demonstrates the certainty of their position. Two viewing angles for each set of residues are shown (left and right).

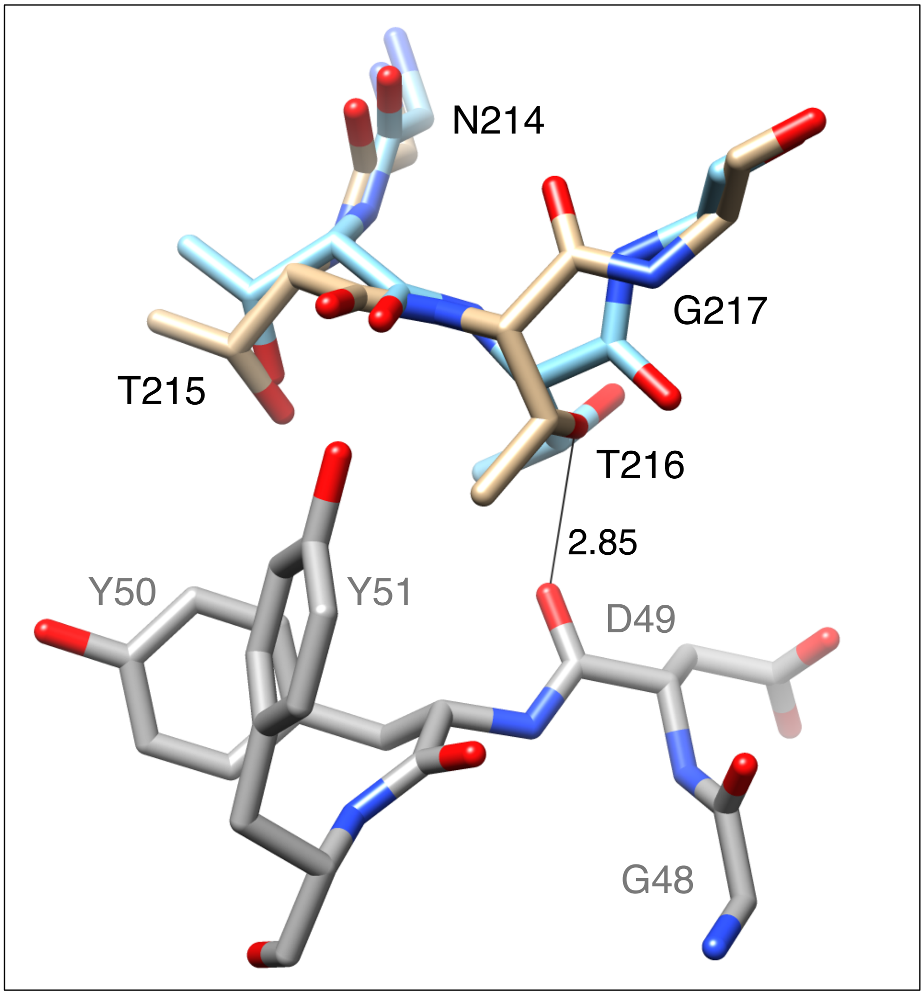

Supplementary Figure 5.
Structure of the BLIP G48-Y51 loop (gray) interactions with the N214-G217 loop region of CTX-M-15 lactamase (tan). The structure of the apo CTX-M-15 N214-G217 loop (cyan) is shown in a structural alignment with the CTX-M-15/BLIP structure. Hydrogen bond is shown as a thin black line and the distance (Å) is indicated. Oxygen is shown in red and nitrogen in blue.

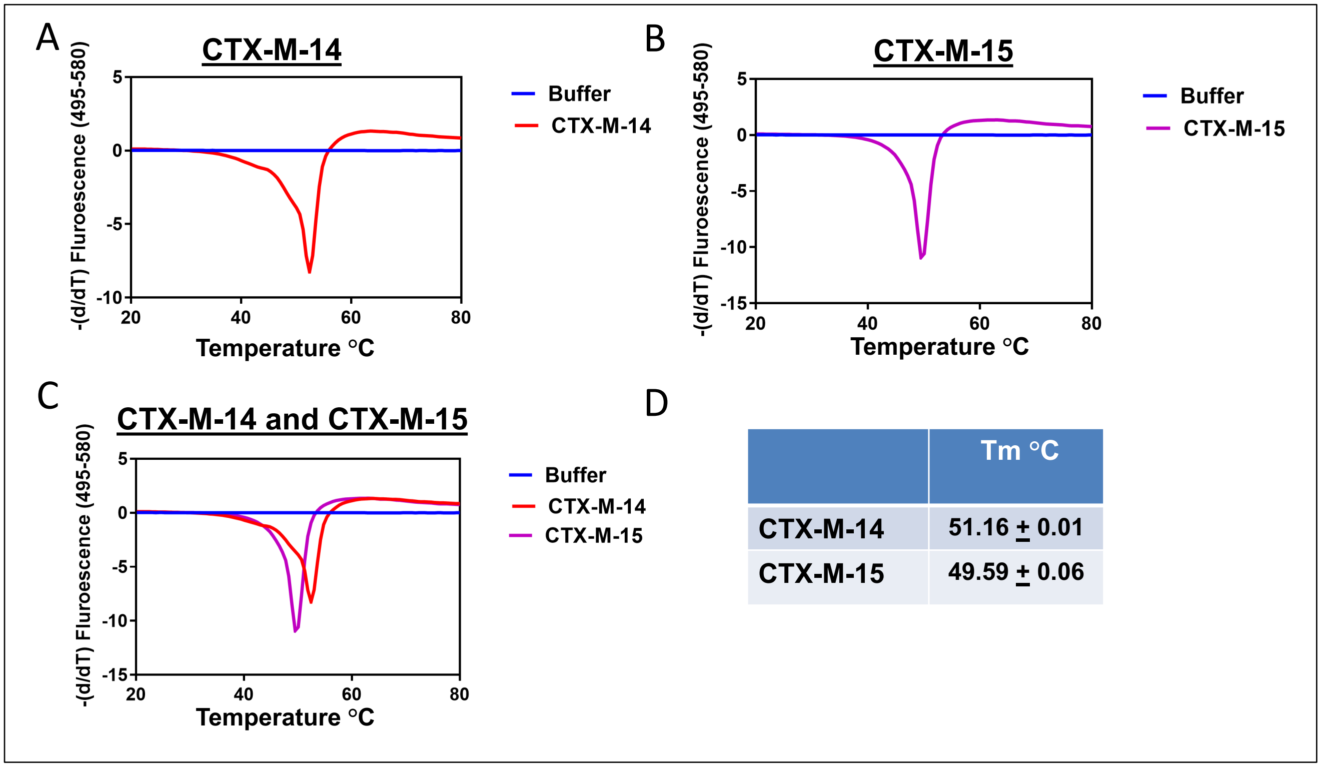

	Tm °C
CTX-M-14	51.16 ± 0.01
CTX-M-15	49.59 ± 0.06

Supplementary Figure 6.
Differential scanning fluorimetry (DSF) measurements of CTX-M-14 and CTX-M-15 β-lactamases thermal stability. **a** DSF scan of CTX-M-14 β-lactamase. **b** DSF scan of CTX-M-15 β-lac tamase. **c** Panel shows the scans for both CTX-M-14 and CTX-M-15. **d** T_m values for CTX-M-14 an d CTX-M-15. Source data are provided as Source data file.

Supplementary Note

Software and Codes for Molecular Dynamics

GROMACS version 2020.6 and the CHARMM36-Jul21 forcefield were used for all the Molecular

Dynamics simulations in this research.

RMSD calculation and distance analysis was performed using GROMACS.

Input structures were downloaded from RCSB PDB database or solved in this research. All the input structures were pre-processed by CHARMM-GUI (https://charmm-gui.org/) Input Generator - PDB Reader module.

Command workflow for GROMACS:

gmx_mpi pdb2gmx -f step1* -o CTX.gro

gmx_mpi editconf -f CTX.gro -o newbox.gro -bt cubic -c -d 1.0

gmx_mpi solvate -cp newbox.gro -cs spc216.gro -p topol.top -o solv.gro

gmx_mpi editconf -f solv.gro -o solv.pdb

gmx_mpi grompp -f ions.mdp -c solv.gro -p topol.top -o ions.tpr

echo "SOL" | gmx_mpi genion -s ions.tpr -o solv_ions.gro -p topol.top -pname NA -nname CL -neutral

gmx_mpi grompp -f em.mdp -c solv_ions.gro -p topol.top -o em.tpr

gmx_mpi mdrun -v -s em.tpr -deffnm em

gmx_mpi grompp -f nvt.mdp -c em.gro -r em.gro -p topol.top -o nvt.tpr

gmx_mpi mdrun -v -s nvt.tpr -deffnm nvt

gmx_mpi grompp -f npt.mdp -c nvt.gro -t nvt.cpt -r nvt.gro -p topol.top -o npt.tpr

gmx_mpi mdrun -v -s npt.tpr -deffnm npt;

gmx_mpi grompp -f md.mdp -c npt.gro -t npt.cpt -p topol.top -o md_CTX.tpr

gmx_mpi mdrun -ntomp 12 -bonded gpu -nb gpu -pme gpu -v -s md_CTX.tpr -deffnm md_CTX -gpu_id 0 -pin on

Trajectories were generated by the following commands:

(echo system)| gmx_mpi trjconv -s md_CTX.tpr -f md_CTX.xtc -o md_CTX_whole.xtc -pbc whole;

(echo protein; sleep 1; echo system)| gmx_mpi trjconv -s md_CTX.tpr -f md_CTX_whole.xtc -o md_CTX3_center.xtc -center -pbc mol -ur compact -n index.ndx;

(echo backbone; sleep 1; echo system)| gmx_mpi trjconv -s md_CTX.tpr -f md_CTX_center.xtc -o md_CTX3_fit.xtc -fit rot+trans;

(echo non-water)| gmx_mpi trjconv -s md_CTX.tpr -f md_CTX_fit.xtc -o CTX_visual.pdb -pbc nojump -dt 100

The parameters in the mdp files used in this research for each MD step is given as follows:

Energy minimization:

title = Minimization ; Title of run

integrator = steep ; Algorithm (steep = steepest descent minimization)

emtol = 1000.0 ; Stop minimization when the maximum force < 10.0 kJ/mol

emstep = 0.01 ; Energy step size

nsteps = 50000 ; Maximum number of (minimization) steps to perform

nstlist = 1 ; Frequency to update the neighbor list and long range forces

cutoff-scheme = Verlet

ns_type = grid ; Method to determine neighbor list (simple, grid)

rlist = 1.2 ; Cut-off for making neighbor list (short range forces)

coulombtype = PME ; Treatment of long range electrostatic interactions

rcoulomb = 1.2 ; long range electrostatic cut-off

vdwtype = cutoff

vdw-modifier = force-switch

rvdw-switch = 1.0

rvdw = 1.2 ; long range Van der Waals cut-off

pbc = xyz ; Periodic Boundary Conditions

DispCorr = no

NVT

title = Protein-ligand complex NVT equilibration

define = -DPOSRES ; position restrain the protein and ligand

; Run parameters

integrator = md ; leap-frog integrator

nsteps = 50000 ; 2 * 50000 = 100 ps

dt = 0.002 ; 2 fs

; Output control

nstenergy = 500 ; save energies every 1.0 ps

nstlog = 500 ; update log file every 1.0 ps

nstxout-compressed = 500 ; save coordinates every 1.0 ps

; Bond parameters

continuation = no ; first dynamics run

constraint_algorithm = lincs ; holonomic constraints

constraints = h-bonds ; bonds to H are constrained

```
lincs_iter              = 1         ; accuracy of LINCS
lincs_order             = 4         ; also related to accuracy
; Neighbor searching and vdW
cutoff-scheme           = Verlet
ns_type                 = grid      ; search neighboring grid cells
nstlist                 = 20        ; largely irrelevant with Verlet
rlist                   = 1.2
vdwtype                 = cutoff
vdw-modifier            = force-switch
rvdw-switch             = 1.0
rvdw                    = 1.2       ; short-range van der Waals cutoff (in nm)
; Electrostatics
coulombtype             = PME       ; Particle Mesh Ewald for long-range electrostatics
rcoulomb                = 1.2       ; short-range electrostatic cutoff (in nm)
pme_order               = 4         ; cubic interpolation
fourierspacing          = 0.16      ; grid spacing for FFT
; Temperature coupling
tcoupl                  = V-rescale                     ; modified Berendsen thermostat
tc-grps                 = Protein Water_and_ions    ; two coupling groups - more accurate
tau_t                   = 0.1   0.1                     ; time constant, in ps
ref_t                   = 300   300                     ; reference temperature, one for each group, in K
; Pressure coupling
pcoupl                  = no        ; no pressure coupling in NVT
; Periodic boundary conditions
pbc                     = xyz       ; 3-D PBC
; Dispersion correction is not used for proteins with the C36 additive FF
DispCorr                = no
; Velocity generation
gen_vel                 = yes       ; assign velocities from Maxwell distribution
gen_temp                = 300       ; temperature for Maxwell distribution
gen_seed                = -1        ; generate a random seed
```

NPT

```
title                   = Protein-ligand complex NPT equilibration
```

```
define                  = -DPOSRES  ; position restrain the protein and ligand
; Run parameters
integrator              = md        ; leap-frog integrator
nsteps                  = 50000     ; 2 * 50000 = 100 ps
dt                      = 0.002     ; 2 fs
; Output control
nstenergy               = 500       ; save energies every 1.0 ps
nstlog                  = 500       ; update log file every 1.0 ps
nstxout-compressed      = 500       ; save coordinates every 1.0 ps
; Bond parameters
continuation            = yes       ; continuing from NVT
constraint_algorithm    = lincs     ; holonomic constraints
constraints             = h-bonds   ; bonds to H are constrained
lincs_iter              = 1         ; accuracy of LINCS
lincs_order             = 4         ; also related to accuracy
; Neighbor searching and vdW
cutoff-scheme           = Verlet
ns_type                 = grid      ; search neighboring grid cells
nstlist                 = 20        ; largely irrelevant with Verlet
rlist                   = 1.2
vdwtype                 = cutoff
vdw-modifier            = force-switch
rvdw-switch             = 1.0
rvdw                    = 1.2       ; short-range van der Waals cutoff (in nm)
; Electrostatics
coulombtype             = PME       ; Particle Mesh Ewald for long-range electrostatics
rcoulomb                = 1.2
pme_order               = 4         ; cubic interpolation
fourierspacing          = 0.16      ; grid spacing for FFT
; Temperature coupling
tcoupl                  = V-rescale                     ; modified Berendsen thermostat
tc-grps                 = Protein Water_and_ions    ; two coupling groups - more accurate
tau_t                   = 0.1   0.1                     ; time constant, in ps
```

```
ref_t                = 300   300                ; reference temperature, one for each group, in K
; Pressure coupling
pcoupl               = Berendsen                ; pressure coupling is on for NPT
pcoupltype           = isotropic                ; uniform scaling of box vectors
tau_p                = 2.0                      ; time constant, in ps
ref_p                = 1.0                      ; reference pressure, in bar
compressibility      = 4.5e-5                   ; isothermal compressibility of water, bar^-1
refcoord_scaling     = com
; Periodic boundary conditions
pbc                  = xyz       ; 3-D PBC
; Dispersion correction is not used for proteins with the C36 additive FF
DispCorr             = no
; Velocity generation
gen_vel              = no        ; velocity generation off after NVT
```

Production run

```
title                = Protein-ligand complex MD simulation
; Run parameters
integrator           = md        ; leap-frog integrator
nsteps               = 50000000   ; 2 * 50000000 = 100000 ps (100 ns)
dt                   = 0.002     ; 2 fs
; Output control
nstenergy            = 5000      ; save energies every 10.0 ps
nstlog               = 5000      ; update log file every 10.0 ps
nstxout-compressed   = 5000      ; save coordinates every 10.0 ps
; Bond parameters
continuation         = yes       ; continuing from NPT
constraint_algorithm = lincs     ; holonomic constraints
constraints          = h-bonds   ; bonds to H are constrained
lincs_iter           = 1         ; accuracy of LINCS
lincs_order          = 4         ; also related to accuracy
; Neighbor searching and vdW
```

```
cutoff-scheme          = Verlet
ns_type                = grid      ; search neighboring grid cells
nstlist                = 20        ; largely irrelevant with Verlet
rlist                  = 1.2
vdwtype                = cutoff
vdw-modifier           = force-switch
rvdw-switch            = 1.0
rvdw                   = 1.2       ; short-range van der Waals cutoff (in nm)
; Electrostatics
coulombtype            = PME       ; Particle Mesh Ewald for long-range electrostatics
rcoulomb               = 1.2
pme_order              = 4         ; cubic interpolation
fourierspacing         = 0.16      ; grid spacing for FFT
; Temperature coupling
tcoupl                 = V-rescale                  ; modified Berendsen thermostat
tc-grps                = Protein Water_and_ions    ; two coupling groups - more accurate
tau_t                  = 0.1   0.1                  ; time constant, in ps
ref_t                  = 300   300                  ; reference temperature, one for each group, in K
; Pressure coupling
pcoupl                 = Parrinello-Rahman          ; pressure coupling is on for NPT
pcoupltype             = isotropic                  ; uniform scaling of box vectors
tau_p                  = 2.0                        ; time constant, in ps
ref_p                  = 1.0                        ; reference pressure, in bar
compressibility        = 4.5e-5                     ; isothermal compressibility of water, bar^-1
; Periodic boundary conditions
pbc                    = xyz       ; 3-D PBC
; Dispersion correction is not used for proteins with the C36 additive FF
DispCorr               = no
; Velocity generation
gen_vel                = no        ; continuing from NPT equilibration
```
