# Supplementary material for: An active site loop toggles between conformations to control antibiotic hydrolysis and inhibition potency for CTX-M β-lactamase drug-resistance enzymes
Source: Nat Commun. 2022 Nov 7;13:6726. doi: 10.1038/s41467-022-34564-3 (PMC9640584; doi:10.1038/s41467-022-34564-3)
Supplement: Supplementary file 3 — Reporting Summary [file 41467_2022_34564_MOESM3_ESM.pdf]

nature portfolio

Corresponding author(s): Timothy Palzkill

Last updated by author(s): Oct 20, 2022

Reporting Summary

Nature Portfolio wishes to improve the reproducibility of the work that we publish. This form provides structure for consistency and transparency in reporting. For further information on Nature Portfolio policies, see our Editorial Policies and the Editorial Policy Checklist.

Statistics

For all statistical analyses, confirm that the following items are present in the figure legend, table legend, main text, or Methods section.

n/a	Confirmed	
☐	☒	The exact sample size (*n*) for each experimental group/condition, given as a discrete number and unit of measurement
☐	☒	A statement on whether measurements were taken from distinct samples or whether the same sample was measured repeatedly
☒	☐	The statistical test(s) used AND whether they are one- or two-sided<br>*Only common tests should be described solely by name; describe more complex techniques in the Methods section.*
☒	☐	A description of all covariates tested
☒	☐	A description of any assumptions or corrections, such as tests of normality and adjustment for multiple comparisons
☐	☒	A full description of the statistical parameters including central tendency (e.g. means) or other basic estimates (e.g. regression coefficient) AND variation (e.g. standard deviation) or associated estimates of uncertainty (e.g. confidence intervals)
☒	☐	For null hypothesis testing, the test statistic (e.g. *F*, *t*, *r*) with confidence intervals, effect sizes, degrees of freedom and *P* value noted<br>*Give P values as exact values whenever suitable.*
☒	☐	For Bayesian analysis, information on the choice of priors and Markov chain Monte Carlo settings
☒	☐	For hierarchical and complex designs, identification of the appropriate level for tests and full reporting of outcomes
☒	☐	Estimates of effect sizes (e.g. Cohen's *d*, Pearson's *r*), indicating how they were calculated

Our web collection on statistics for biologists contains articles on many of the points above.

Software and code

Policy information about availability of computer code

Data collection	No software was used.
Data analysis	Enzyme kinetics/inhibition data was analyzed with GraphPad Prism 9.0. X-ray diffraction data was analyzed with Coot v1, HKL200 v722, iMosflm v7.4, in the CCP4 v7.1 suite, Phaser v2.1.2, Phenix v1.17.1, which are open source and cited in the manuscript. Structure analysis and figures were made with Chimera v1.13.1 and ChimeraX v1.4, Ligplot+ v2.2.4 and cited in manuscript. GROMACS v2020.6 and the CHARMM36-Jul21 forcefield were used for MD simulations as cited. The sequence alignment of CTX-M-14 and CTX-M-14 beta-lactamases was performed using Clustal Omega using the EMBL-EBI web server.

For manuscripts utilizing custom algorithms or software that are central to the research but not yet described in published literature, software must be made available to editors and reviewers. We strongly encourage code deposition in a community repository (e.g. GitHub). See the Nature Portfolio guidelines for submitting code & software for further information.

Data

Policy information about availability of data

All manuscripts must include a data availability statement. This statement should provide the following information, where applicable:

- Accession codes, unique identifiers, or web links for publicly available datasets
- A description of any restrictions on data availability
- For clinical datasets or third party data, please ensure that the statement adheres to our policy

The BLIP/CTX-M-15 structure information has been deposited in the Protein Data Bank under accession code PDB id: 7S5S [http://doi.org/10.2210/pdb7S5S/pdb].

The enzyme inhibition data and the differential scanning fluorimetry data is available as supplemental data file Source-data. The command workflow for the molecular dynamics simulations is available as supplemental information file Supplementary-Data-MD-simulations. The molecular dynamics trajectories have been deposited at Figshare [http://doi.org/10.6084/m9.figshare.21378981]. Plasmid expression constructs of BLIP and beta-lactamases are available upon request.

Human research participants

Policy information about studies involving human research participants and Sex and Gender in Research.

Reporting on sex and gender	N/A
Population characteristics	N/A
Recruitment	N/A
Ethics oversight	N/A

Note that full information on the approval of the study protocol must also be provided in the manuscript.

Field-specific reporting

Please select the one below that is the best fit for your research. If you are not sure, read the appropriate sections before making your selection.

☒ Life sciences ☐ Behavioural & social sciences ☐ Ecological, evolutionary & environmental sciences

For a reference copy of the document with all sections, see nature.com/documents/nr-reporting-summary-flat.pdf

Life sciences study design

All studies must disclose on these points even when the disclosure is negative.

Sample size	Enzyme inhibition kinetics were performed a minimum of two times. Additional trials were performed if the standard error was above 20% of the value.
Data exclusions	No data exclusions.
Replication	Enzyme kinetics experiments to determine the Km for the reporter substrate nitrocefin were performed by two individuals with separate enzyme preparations. The standard deviation upon replication in the Km determinations was <20%. The inhibition assays with purified BLIP were performed a minimum of two times as described in the sample size section above.
Randomization	Randomization was not performed in enzyme kinetics/inhibition studies as the reaction conditions including enzyme and inhibitor concentration and range of inhibitor used are dependent on the individual protein involved in the experiment.
Blinding	Blinding was not performed. It is not possible to blind X-ray structure determination. Blinding was not performed in enzyme kinetics/inhibition studies as the reaction conditions including enzyme and inhibitor concentration and range of inhibitor used are dependent on the individual protein involved in the experiment.

Reporting for specific materials, systems and methods

We require information from authors about some types of materials, experimental systems and methods used in many studies. Here, indicate whether each material, system or method listed is relevant to your study. If you are not sure if a list item applies to your research, read the appropriate section before selecting a response.

Materials & experimental systems

n/a	Involved in the study	
☒	☐	Antibodies
☒	☐	Eukaryotic cell lines
☒	☐	Palaeontology and archaeology
☒	☐	Animals and other organisms
☒	☐	Clinical data
☒	☐	Dual use research of concern

Methods

n/a	Involved in the study	
☒	☐	ChIP-seq
☒	☐	Flow cytometry
☒	☐	MRI-based neuroimaging
